# Effect of Enzymatic Biotransformation on the Hypotensive Potential of Red Grape Pomace Extract

**DOI:** 10.3390/foods12224109

**Published:** 2023-11-13

**Authors:** Daniel Batista, Gabriela de Matuoka e Chiocchetti, Juliana Alves Macedo

**Affiliations:** Department of Food Science and Nutrition, School of Food Engineering, University of Campinas, Campinas 13083-862, SP, Brazil; gmchiocchetti@gmail.com (G.d.M.e.C.); jumacedo@unicamp.br (J.A.M.)

**Keywords:** agricultural residues, phenolic compounds, biotransformation, angiotensin-converting enzyme, hypertension

## Abstract

Hypertension is a widespread health risk, affecting over a billion people and causing 9 million deaths per year. The Renin–Angiotensin–Aldosterone System (RAAS) is a primary target for hypertension treatment, and it is primarily treated through drugs that inhibit the Angiotensin I-Converting Enzyme (ACE). In addition to pharmacological treatment, various plants are recommended in traditional medicine for blood pressure regulation. This study aimed to produce high-phenolic-content extracts with and without enzymatic assistance from red grape pomace and evaluate their antioxidant capacity and ACE inhibitory potential. The total phenolic content (TPC) was measured, and phenolic identification was performed using HPLC analysis. In addition, the antioxidant capacity and anti-hypertensive potential were determined via in vitro assays. There was no statistical difference in the TPC antioxidant capacity between the extraction methods. Otherwise, when considering the extraction yield, the enzymatic process recovered around 70% more phenolic compounds from the pomace, and the phenolic profile was changed. Enzymatic assistance also significantly increased the ACE inhibitory potential in the grape pomace extract. This study demonstrates the viability of upcycling grape pomace to obtain bioactive compounds and to reduce their environmental impact, and highlights the influence of the enzymatic extraction on the hypotensive potential of the extract.

## 1. Introduction

Hypertension is characterized by the occurrence of blood pressure (BP) with a value equal to or higher than 140 mmHg for systolic pressure or 90 mmHg for diastolic pressure (140/90 mmHg), and affects more than one billion people around the world [1]. High blood pressure results in the heart exerting greater effort to distribute blood into the body and is considered a risk factor for the occurrence of cardiovascular diseases such as stroke and heart attack. This fact means that hypertension poses a great challenge to public health, with approximately nine million deaths per year being attributed to complications caused by the disease, according to previously published reports [2,3].

The first medicine developed for hypertension was captopril, a strong inhibitor of the Angiotensin-Converting Enzyme (ACE) that breaks the Renin–Angiotensin–Aldosterone System (RAAS) cascade reaction [4,5]. The RAAS is one of the most important mechanisms for physiological BP control, acting via the release of vasoconstrictors and the reabsorption of sodium and water, which leads to the elevation of BP in cases of hypotensive episodes; in addition, its deregulation is one of the mechanisms involved in the occurrence of hypertension. As such, the most popular treatments for hypertension, such as captopril, act mainly on the inhibition of the enzymes and receptors involved in the RAAS cascade reaction. Besides pharmacological treatment, it is possible to find, in popular medicine, recommendations for the utilization of different plants, fruits, and herbs for BP control [6,7,8]. Many studies have been published correlating the hypotensive potential of some plants and/or food extracts rich in phenolic compounds with their ability to modulate the RAAS, mainly via ACE inhibition [9,10,11,12]; this is having shown a positive correlation between the content of phenolics, including flavonoids, and the in vitro potential of the enzyme for inhibition [9,13,14]. The correlation between in vitro hypotensive potential and in vivo and ex vivo studies was confirmed by Mao et al. [15]; using an extract with a constant total phenolic content (50 μg/mL of gallic acid equivalent), their results showed that the phenolic extract was able to relax the aortic vessel in an ex vivo experiment and bring the BP of Wistar rats, with hypertension induced by sodium chloride, to normal parameters.

Polyphenols are secondary metabolites found in plants and are recognized due to their antioxidant activity, among other health benefits. However, to obtain these benefits, a high consumption of fruits and vegetables is necessary, as these are a source of such compounds. Studies have shown that the extent of polyphenol intake varies worldwide, mainly because of different population dietary habits, and economic access to fresh healthy food. Studies have shown that, in Europe, the consumption of polyphenols ranges from 820 to 1741 mg/day, while in Brazil, this value ranges from 377 to 460 mg/day [16]. For the United States of America, considering the consumption of the most common fruits and vegetables, the estimated consumption of polyphenols is 455.7 mg of gallic acid equivalent per day, with oranges, apples, and potatoes being the biggest contributors [17]. An alternative able complement the daily consumption of polyphenols could be supplemented or fortified industrial food, obtained from low-cost and high-viability sources of bioactive compounds, such as agro-industrial residues; these are the residues of fruits and vegetables from the processing of certain foods, like juice, wine, or frozen foods.

Tons of these residues are produced annually from food processing, configuring an environmental problem for the industry [18,19]. On the other hand, there is increasing interest in upcycling studies addressing the utilization of the natural compounds present in these residues as functional ingredients in the food industry, with the aim being to increase the nutritional and technological profile of products. In this context, grape pomaces from wine production have a large availability and are recognized as a great source of phenolic compounds. Viticulture is one of the world’s largest fruit cultures, with a production of more than 77 million tons in 2019; in addition, approximately 75% of grape production is destined for winemaking. Residues from wineries correspond to around 20 to 30% of the weight of the fresh fruit, depending on the utilized process [20,21]. Some studies have suggested that red grape pomace is a good source of dietary fiber, polyunsaturated fatty acid, and phenolic compounds, which has aroused interest regarding the potential utilization of this residue in obtaining high-value-added products [20,22,23,24].

Different processes can be used for the extraction of bioactive compounds. Biotransformation consists of the utilization of cells, microorganisms, or isolated enzymes for promoting alterations in the chemical structure to improve compounds’ bioavailability or bioactivity [25]. Among the biotransformation methods, enzymatic treatment is of great interest, as it can increase the extraction of compounds in plants, breaking the cell walls of the matrix and releasing the compounds linked to it, as well as increasing their biological properties via structural modification and enhancing the absorption or biological activity [26]. Naturally, polyphenols are present in agro-industrial wastes in their glycosylated, ester or polymer form, which makes their absorption and metabolism difficult; thus, their aglycone forms present greater benefits to human health [25,27]. Different enzymes, such as β-glucosidase (EC 3.2.1.21), pectinase (EC 3.2.1.15), and cellulase (EC 3.2.1.4), are used in the food industry to improve the production rate or enhance food’s technological or nutritional properties [28]. In addition, several studies have demonstrated the benefits of carrying out enzymatic treatments to obtain phenolic extracts with greater biological potential. Ruviaro et al. [29] investigated the effect of using different enzymes (isolated or combined) for an extract obtained from citrus byproducts. The results showed an improvement in the antioxidant capacity and antihypertensive potential of extracts when enzymatic treatment was used. Chamorro et al. [30] also evaluated the effect of enzymatic treatment (carbohydrates with cellulolytic and pectinolytic activities and tannase) on the antioxidant activity and solubilization of sugars in grape seed and pomace extracts. Tannase treatment increased the total phenolic content, gallic acid, epicatechin, and procyanidin B2 in the seed extract, with an increase in antioxidant activity being observed; an even greater effect was observed when tannase and pectinase were used in combination.

Based on the aforementioned, this work aimed to evaluate the antioxidant capacity and hypotensive potential (by ACE inhibition) of enzymatic-assisted phenolic extracts obtained from red grape agro-industrial residues. The control extract obtained without enzyme assistance was evaluated in all the assays for comparison purposes.

## 2. Materials and Methods

### 2.1. Chemicals

Gallic acid, quercetin, Trolox^®^, fluorescein, procyanidin B2, Catechin, caffeic acid, rutin, Coomassie Blue Brilliant G-250, 2,2′azobis-(1-methylpropionamidine)-dihydrochloride (AAPH), tripyridyltriazine (TPTZ), 2,2-diphenyl-1-picrylhydrazyl (DPPH), ρ-nitrophenyl-β-glycoside (N7006), naringinase from *Penicillium decumbers* (N1385), the Angiotensin I-Converting Enzyme Activity Assay Kit (CS0002), captopril (PHR1307) and galacturonic acid were purchased from Sigma-Aldrich (St. Louis, MO, USA). Folin–Ciocalteu reagent, monobasic and dibasic sodium phosphate, sodium carbonate, formic acid and 3,5-dinitrosalicyl acid were purchased from Dinâmica Química Contemporânea (Diadema, SP, Brazil). Celluclast^®^ 1.5 L and Pectinex^®^ Ultra SP-L enzymes (Novozymes, Bagsværd, Demark) were donated by LNF Latino América (Bento Gonçalves, RS, Brazil). All other reagents were of analytical grade.

### 2.2. Samples and Enzymes

Red grape pomace (75% Syrah and 25% Seibel), constituted by the grape skin, seeds, and stems, as well as the natural microbiota of the fruit derived from fermentation during wine production, was obtained from wineries in Rio Grande do Sul state, Brazil, collected during January and March 2013. The residue was ground with a portable laboratory grinder (Blender OBL10/2, Machesoni, Sao Paulo, Brazil), freeze-dried, and stored under −80 °C in a vacuum pack until extraction. For enzymatic extraction, Celluclast^®^ 1.5 L and Pectinex^®^ Ultra SP-L from Novozymes were used, with enzymatic activity expressed in U/mL, and naringinase from *Penicillium decumbers* from Sigma-Aldrich was used, with β-glucosidase activity expressed in U/g. One enzymatic unit (U) was considered the amount of enzyme needed to release 1 µmol of enzymatic product from the substrate per minute (µmol/min).

Cellulase activity was determined using the dinitrosalicylic acid (DNS) method, according to Wood and Garcia-Campayo [31], with modification. Briefly, 0.45 mL of 1% carboxymethylcellulose solution in 5 mM of citrate buffer (pH 4,8) was acclimatized at 40 °C for 10 min; then, it was added to 0.05 mL of enzyme solution (1:500 to 1:9000), followed by 60 min incubation at 40 °C. After incubation, 0.5 mL of DNS reagent was added, followed by a boiling bath and an ice bath for 5 min each, and 4 mL of distilled water was added. For the calibration curve, 0.5 mL of glucose in the reaction buffer (0.56 to 5.6 μmol/mL) was used, and the steps above were followed for DNS addition. Absorption was read at a 540 nm wavelength, using a 96-well plate in a plate reader (ST-360, KHB, Shanghai, China).

Pectinase activity determination was carried out using the DNS method as proposed by Couri [32], with modifications. Briefly, 2 mL of 2.5% polygalactoronic acid solution in 20 mM of acetate buffer (pH 5) was acclimatized in a 40 °C bath for 10 min; then, 0.05 mL of enzyme solution was added (1:500 to 1:9000), followed by incubation for 30 min at 40 °C. After incubation, 0.05 mL of this solution was transferred to tubes containing 0.5 mL of DNS reagent, followed by the addition of 0.45 mL of distilled water and boiling bath for 5 min. After that, tubes were put in an ice bath, and 4 mL of distilled water was added. The calibration curve was plotted using 0.05 mL of galacturonic acid in reaction buffer (0.45 to 5.6 μmol/mL), adding it in tubes and following the steps used above for DNS addition. Absorption was read at a 540 nm wavelength, using a 96-well plate in a plate reader (ST-360, KHB, Shanghai, China).

β-glucosidase activity was determined according to the method proposed by Matsuura, Sasaki, and Murao [33], with modifications by Ávila et al. [27]. First, 0.3 mL of a 5 mM ρ-nitrophenyl-β-glycoside solution in 0.1 M acetate buffer (pH 5) was acclimatized in a 40 °C bath for 10 min, followed by the addition of 0.3 mL of enzyme solution (0.01 to 0.1 mg/mL) and incubation for 30 min at the same temperature. The reaction was stopped by adding 0.3 mL of 0.5 M sodium carbonate solution. The calibration curve was plotted using ρ-nitrophenol solution (0.009 to 0.216 μmol/mL), reaction buffer, and sodium carbonate solution. Absorption was read at a 410 nm wavelength, using a 96-well plate in a plate reader (ST-360, KHB, Shanghai, China).

### 2.3. Extracts Preparation

The extraction protocol was adapted from Martins et al. [24] and Xu et al. [34]. An enzymatic blend containing 1000 U of cellulase, 100 U of pectinase, and 250 U of β-glucosidase in 100 mL of 0.1 M acetate buffer (pH 5) solution was acclimated in a 50 °C water bath for 10 min; then, 10 g of sample was added and incubated in a stirring bath (Tecnal TE-053, Limeira, Brazil) under 130 rpm agitation and 50 °C for 5 h. After that, 100 mL of absolute ethanol was added, resulting in a 1:1 (*v*/*v*) ethanol/buffer solution. After incubation for 50 min more, in the same condition of agitation and temperature, the samples were sonicated in an ultrasonic bath (USC-1800A, Unique, Indaiatuba, Brazil) for 10 min. The supernatant was separated via centrifugation at 5.500 rpm for 15 min (Megafuge 16R, Thermo Scientific, EUA, Waltham, MA, USA), followed by vacuum filtration. Non-enzymatic extraction (control) was conducted as above, replacing the enzymatic solution with the 0.1 M acetate buffer (pH 5). Ethanol was removed from the extracts using a rotary evaporator (W240, Büchi, Flawil, Switzerland) at 50 °C. The resultant extracts were frozen and freeze-dried (Lyophilizer Liotop L101, Liobras, São Carlos, Brazil), and stored at −20 °C until utilization. Two phenolic extracts were prepared: enzymatic and non-enzymatic extraction from red grape pomace (EGP and GP, respectively). The extraction yield was calculated by dividing the mass of the dried extract by the mass of dried sample, and expressed as a percentage (%).

### 2.4. Total Phenolic Content

The Total Phenolic Content (TPC) was determined using the Folin–Ciocalteu reagent as described by Singleton and Rossi [35], with modifications. Into a 96-well plate, 15 μL of extract solution, 240 μL of distilled water, and 15 μL of Folin–Ciocalteu reagent were added; after 3 min, 30 μL of the 10% sodium carbonate solution was added and kept in the dark for 2 h. For the calibration curve, the extracts were replaced by gallic acid solution (16 to 300 μg/mL). Absorbance was read at a 760 nm wavelength in a plate reader (ST-360, KHB, Shanghai, China), and the results were expressed as milligrams of gallic acid equivalent per gram of dried extract (mg GAE/g DE).

### 2.5. Phenolic Identification by HPLC Analysis

The polyphenolics in the extracts were quantified according to Martins et al. [24] using a Dionex UltiMate 3000 (Dreieich, Germany) HPLC system with a RP18 Waters X-Terra^®^ (Milford, USA) column (5 μm, 4.6 × 150 mm) that was maintained at 30 °C, and a UV/VIS detector (DAD-3000, Dreieich, Germany). Analyte separation was achieved using mobile phase A (water/formic acid, 99.9:0.1 *v*/*v*) and mobile phase B (methanol/formic acid, 99.9:0.1 *v*/*v*) in a linear gradient mode (% solvent A): 92% (0–5 min), 92–85% (5–13 min), 85–75% (13–45 min), 75–57% (45–67 min), 57–50% (67–77 min), 50–35% (77–95 min), 35–20% (95–108 min), 20–92% (108–110 min), 92% (110–120 min). The flow rate was set at 0.5 mL/min. The identification of individual compounds was achieved by comparing their retention time and spectrum. Gallic acid, catechin, resveratrol, and procyanidin B2 were detected at 280 nm, rutin and quercetin were detected at 260 nm, and caffeic acids were detected at 310 nm. Compounds were quantified using standard curves constructed using the authenticated standards.

### 2.6. Antioxidant Capacity

The DPPH reduction assay was performed according to the method proposed by Peschel et al. [36], with modifications by Macedo et al. [37]. First, 50 μL of 70% methanol extract solution was incubated with 150 μL of 0.2 mM DPPH methanol solution in a 96-well plate. For the calibration curve, Trolox solution was used (3.8 to 76 μg/mL). The absorbance change was measured for 75 min at a 520 nm wavelength using a fluorimeter (FLUOstar Optima, BMG Labtech, Ortenberg, Germany).

The oxygen radical absorbance capacity (ORAC) assay was carried out according to the method proposed by Ou et al. [38] and Macedo et al. [37]. First, 20 μL of the extract solution was incubated with 120 μL of 70 nM fluorescein solution in a 96-well black plate, followed by the addition of 60 μL of 12 mM AAPH solution immediately before the reading was initiated. All solutions were prepared in 7.5 mM of potassium phosphate buffer (pH 7.4) and protected from light. The calibration curve was plotted using Trolox solution (0.95 to 950 μg/mL). The fluorescence was monitored in a fluorimeter (FLUOstar Optima, BMG Labtech, Ortenberg, Germany) at 37 °C for 98 min (reading every 75 s), with an excitation filter at 485 nm and an emission filter at 520 nm. The results were calculated considering the area under the curve (AUC).

The ferric reducing antioxidant power (FRAP) assay was performed according to the method proposed by Benzie and Strain [39], with modification. The FRAP reagent solution was prepared by mixing 2.5 mL of 0.3 M acetate buffer (pH 3.6), 250 μL of 10 mM tripyridyltriazine (TPTZ) in 40 mM of HCl solution, and 250 μL of 20 mM ferric chloride solution. Then, 36 μL of the extract solution was incubated with 270 μL of the FRAP reagent in a 96-well plate. For the calibration curve, Trolox solution was used (1.5 to 100 μg/mL). Absorbance was read at 595 nm for 10 min (7 cycles of 88 s) using a fluorimeter (FLUOstar Optima, BMG Labtech, Ortenberg, Germany).

Results were expressed as micromol of Trolox equivalent per gram of dried extract (μmol TE/g DE).

### 2.7. In Vitro ACE Inhibitory Activity

This method is based on the capacity of ACE to hydrolyze a synthetic fluorogenic peptide, releasing a fluorescent compound, and was adapted from the Technical Bulletin of ACE Activity Assay Kit [40]. First, 25 μL of the extract (10 μg GAE/mL) or captopril (50 ng/mL) solution was incubated with 25 μL of the kit enzyme solution (CS0002B) in a 96-well black plate for 5 min at 37 °C. The reaction was started by adding 50 μL of substrate solution (CS0002C), and the fluorescence change was monitored using a fluorimeter (FLUOstar Optima, BMG Labtech, Ortenberg, Germany) at 37 °C each minute for 5 min, with an excitation filter at 340 nm and an emission filter at 405 nm. For the determination of the ACE positive control activity, the extracts were replaced by the assay buffer. The standard curve was plotted using a kit standard solution (CS0002D). All reagents were prepared in the assay buffer (CS0002A) at the recommended dilution immediately before performing the assay. The obtained data (in duplicate) were treated using the Excel-based calculation sheet made available by the producer in order to obtain ACE activity. The results were expressed in the percentage of ACE inhibition and calculated using Equation (1).

(1) $$\%ACEi=\left(\frac{PC-Sp}{PC}\right)\times 100$$

where %*ACEi* is the percentage of *ACE* inhibition, *PC* is the *ACE* activity in the positive control (without inhibitors), and *Sp* is the *ACE* activity in the samples (extracts or captopril). One unit of *ACE* was defined as the amount of enzyme needed to release 1 nmol of the fluorescent compound from the substrate per minute, under assay conditions.

### 2.8. Statistical Analysis

The measurements were performed in triplicate and the results were expressed as mean ± standard deviation (SD). The statistical differences between groups were measured using one-way analysis of variance (ANOVA) and Tukey’s test was used for post hoc comparisons. Differences were considered significant when *p* ≤ 0.1. Statistical analyses were performed using jamovi software version 1.6.23 [41] and GraphPad Prism version 8.0.

## 3. Results and Discussion

### 3.1. Effect of Enzymatic Treatment on Extraction Yield and TPC

The TPC values obtained for the control (GP) and enzymatic (EGP) grape pomace extract were not statistically different, as shown in Table 1. However, enzyme assistance presented an increase of 1.6× compared to the yield extraction; therefore, when the TPC per gram of dried sample is considered (correlating the found TPC in the extract with the extraction yield), it is possible to observe that, although enzymatic extraction did not present a good result regarding an increase in the TPC of the dried extract, the content of phenolic compounds extracted from the residues using enzymes was 72% higher when compared to their non-enzymatic extraction, which is important when considering the total amount of phenolics extracted from the food matrix.

Interest in enzymatic treatment is based on its potential to improve the phenolic extraction rate via the liberation of the compound from the pattern, as well as to improve compounds’ biological activity via structural alterations [26]. In this work, the treatment of the grape pomace with commercially available enzymes increased the yield value, in comparison to the extract without enzymes. This can be explained by cellulase and pectinase activities. Cellulase catalyzes cellulose hydrolysis, while pectinase has the same effect on pectin; the combination of these enzymes during the extraction process can propitiate the higher release of different compounds bound to the vegetal matrix, increasing extraction yields. However, it does not represent a higher content of TPC in the obtained extracts in this work (Table 1). Previous studies using tannase crude enzyme extract for red grape pomace extraction reported an increase in TPC of 39% in the dried extract [42], indicating that this enzyme extract could be more effective in obtaining extracts with increased TPC levels in the vegetable matrix than the commercial enzymes utilized in this work. This effect can be explained by the high tannase activity in this crude enzymatic extract, which acts mainly on ester or terminal bounds in tannins and catechin gallates, promoting the release of phenols without increasing the liberation of other compounds from the matrix [37,43]. On the other hand, investigating commercially available enzymes, as we did in this work, is important since at this tannase crude extract was made by our research group and is not yet available for industrial purposes.

### 3.2. Phenolic Identification by HPLC Analysis

**Figure 1:**
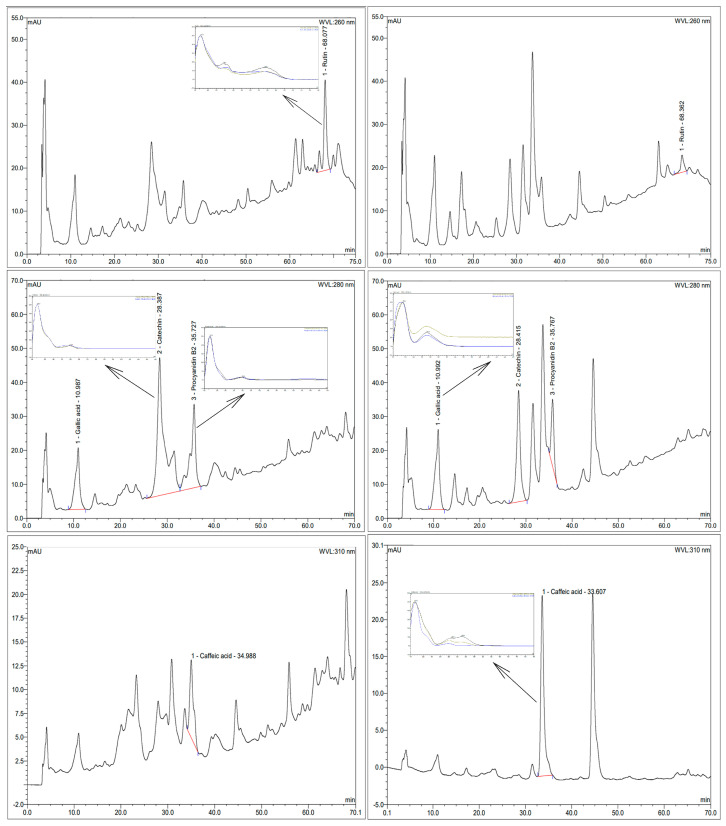
Chromatograms of extracts and UV profile of detected polyphenols. A and B: rutin at 260 nm; C and D: gallic acid, catechin and procyanidin B2 at 280 nm; E and F: caffeic acid at 310 nm. A, C, and E: control extract. B, D, and F: enzymatic extract. Extracts were at a concentration of 20 mg/mL, except in F, which was used at a concentration of 5 mg/mL.

**Figure 2:**
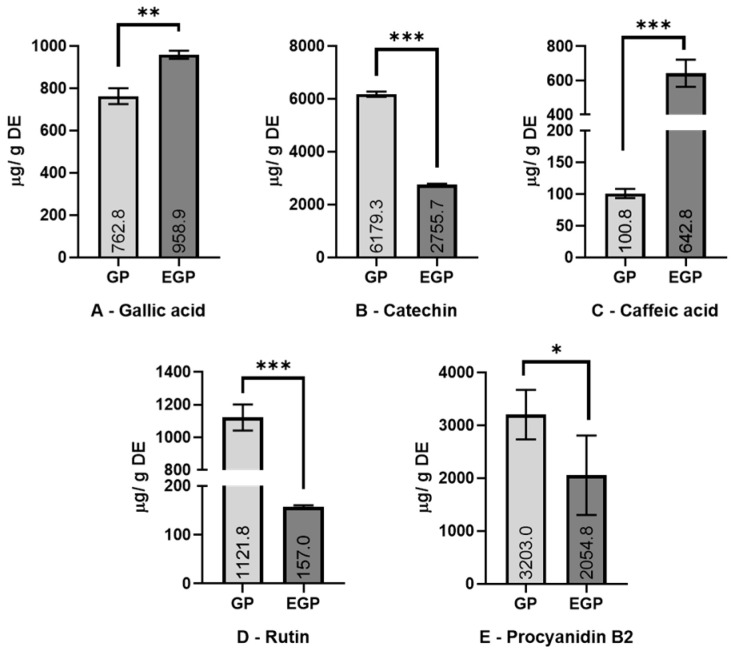
Content of detected polyphenols (A—gallic acid; B—catechin; C—caffeic acid; D—rutin; E—procyanidin B2). Results are expressed as mean ± standard deviation (*n* = 3). Values are expressed as μg/g of dried extract (DE). Extracts: GP—grape pomace; EGP—enzymatic grape pomace. Significantly statistical difference: * *p* < 0.1; ** *p* < 0.005; *** *p* < 0.001.

Nine authenticated standard compounds were used for the construction of the standard curves, but quercetin, resveratrol, epigallocatechin, and epigallocatechin gallate were not detected in the samples. The chromatograms of the extracts and the UV profile of the detected polyphenols are presented in Figure 1, while their contents are presented in Figure 2.

Enzymatic assistance increased the content of gallic acid by 25% and caffeic acid by more than 5 times. Meanwhile, a decrease was observed in the amount of catechin (55%), rutin (86%), and procyanidin B2 (36%) present. The increase in phenolic acids (caffeic and gallic) in the enzymatic extract might be related to the activity of pectinase and cellulase enzymes, which could promote the liberation of these compounds from the matrix; meanwhile, the decrease in rutin (quercetin-3-O-rutinoside), as is observed in this work, might be due the promotion of deglycosylation by β-glucosidase. It was thought that the amount of rutin reduced in the EGP would be present as quercetin (aglycone form of rutin), but possibly as quercetin-3-glucoside, losing only one sugar molecule of rutinose (6-O-α-l-rhamnosyl-d-glucose); however, this was not detected via the HPLC assay. The reduction observed in the amounts of catechin and procyanidin can be associated with the oxidation of those compounds during the enzymatic extraction process. Except for gallic and caffeic acids, the results are different from those found by Martins et al. [24], who used a combination of tannase, cellulase, and pectinase enzymes; meanwhile, this work uses β-glucosidase to replace the tannase crude extract, which can partially explain the differences.

### 3.3. Antioxidant Capacity of Extracts

The values for the DPPH, ORAC, and FRAP assays are presented in Table 2. In general, there was no significant difference between the antioxidant capacity results for the extract obtained using the enzymatic-assisted process and its control, considering the different radicals assessed. Considering that there was no statistical difference in TPC between those extracts, this result was expected.

The results of the DPPH assay for GP and EGP were lower than those reported by Martins et al. [42] for grape pomace extracts with and without enzymatic treatment (3382 and 5309 µmol TE/g DE, respectively); however, the authors reported a higher TPC for their extract as well. The ORAC values for GP and EGP were similar to those reported by Rasines-Perea et al. [44], who evaluated extracts of different red grape species and found values between 1033 and 2614 μmol TE/g. Concerning this, the use of these enzymes to increase the antioxidant capacity of the extracts obtained from this residue under the extraction conditions tested had no significant effect, although the utilization of enzymes could increase the extraction yield.

**Figure 3:**
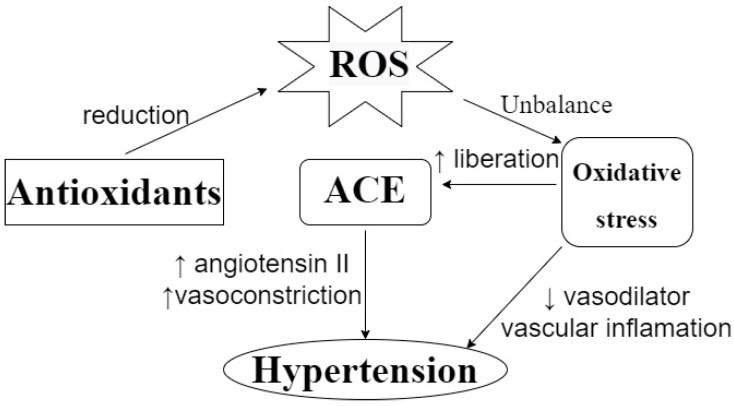
Effect of ROS on blood pressure and the action of antioxidants to prevent it. ROS: Reactive Oxygen Species. ACE: Angiotensin-Converting Enzyme. ↑: increasing. ↓: decreasing. ROS unbalance can lead to oxidative stress, which can promote ACE liberation, vascular inflammation, and a reduction in vasodilators, leading to a hypertensive condition. Antioxidants can prevent this by reducing circulating ROS levels.

Antioxidant activity is important in the etiology of several chronic diseases, including cardiovascular diseases. A study carried out in Spain analyzed the effect of the Mediterranean Diet on the prevention of cardiovascular diseases, and showed that the consumption of 820 ± 323 mg/day of polyphenols, considering flavonoids and phenolic acids, is correlated with the prevention of cardiovascular diseases [45]. More specifically, it has also been found that antioxidant activity can contribute to controlling hypertension by reducing the circulation of reactive oxygen species (ROS). As can be seen in Figure 3, the dysregulation of the oxidative status in the human body can lead to oxidative stress, which can be correlated with hypertension and arteriosclerosis. The effects of oxidative stress include a reduction in the bioavailability of vasodilators, the promotion of vascular inflammation, and an increase in the release of ACE, which can affect blood pressure [46,47,48]. Da Costa et al. [49] evaluated the antihypertensive effect of a phenolic extract of *Euteroe oleracea* Mart. in rats, and indicated that antioxidant capacity is one of the action mechanisms involved in this effect; meanwhile, Ahmad et al. [46] suggested that the possible antihypertensive effect of polyphenols can be attributed to their action as a metal chelator, their free radical scavenging capacity or their stimulation of endogenous antioxidant enzymes.

Although the use of maceration enzymes did not have the effect of producing extracts with a greater antioxidant potential, both extracts obtained in this study showed great levels of antioxidant activity, with a potential for contributing to the control of blood pressure via this mechanism.

### 3.4. In Vitro ACE Inhibitory Activity

**Figure 4:**
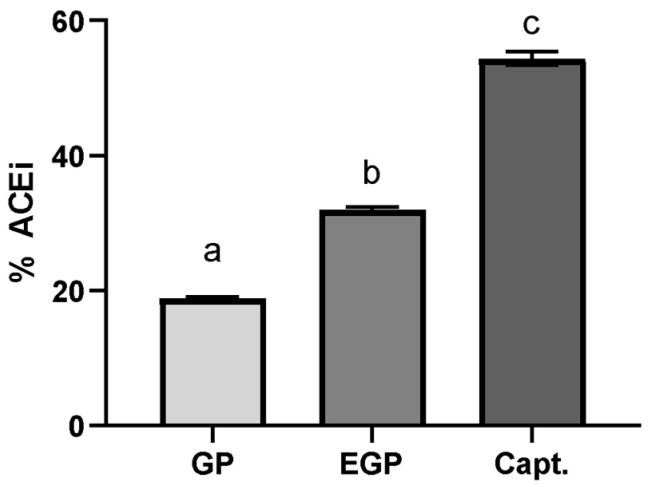
Percentage of ACE inhibition using extract solutions at 10 μg of GAE/mL and captopril solution at 50 ng/mL. %ACEi = 18.8 ± 0.0 for GP, 31.8 ± 0.5 for EGP and 54.4 ± 1.0 for captopril. Different letters mean significant differences according to Tukey’s test (*p* ≤ 0.1).

The samples were evaluated at a determined equivalent gallic acid content (10 μg GAE/mL); this option aimed to standardize the total phenolic content in the sample used, in order to compare its ACE inhibition potential. The results are shown in Figure 4. captopril was used as a standard inhibitor at a concentration of 50 ng/mL [50,51].

**Figure 5:**
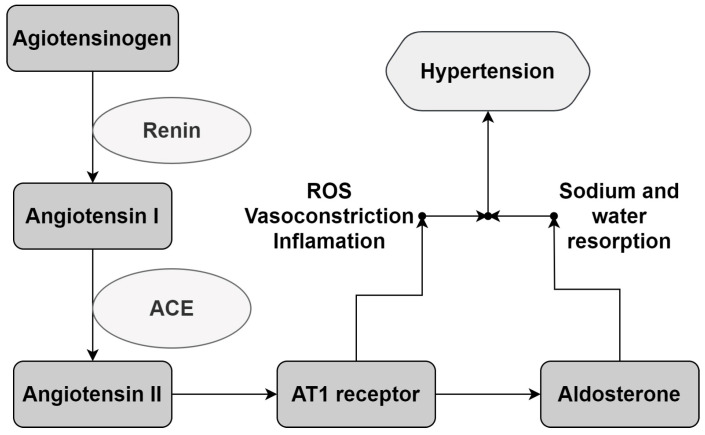
The RAAS reaction cascade. Renin promotes Angiotensin I liberation from angiotensinogen. Angiotensin I is converted to Angiotensin II by the Angiotensin I-Converting Enzyme (ACE). The biological response occurs via the interaction of Angiotensin II with its receptors, promoting vasoconstrictor and aldosterone secretion, which sends signals to the kidneys for water and sodium resorption, leading to a rise in BP. AT1: Angiotensin II receptor type 1. ROS: Reactive Oxygen Species. Adapted from Victor and Kaplan [5].

The ACE inhibitory potential was found to be correlated with BP control via its effect on the RAAS. This system is one of the most important mechanisms for the regulation of cardiovascular and renal function, being activated mainly in episodes of hypovolemia and hypotension. During these episodes, the system promotes vasoconstriction and the resorption of sodium and water by the kidneys. The reregulation of the RAAS due to genetic or environmental factors can promote a continuous and dysregulated rise in blood pressure, leading to hypertension [4,5]. Figure 5 describes the reaction cascade involved in the increase in BP via the RAAS.

Due to its significance in BP regulation, the RAAS is the most important target for hypertension treatment, mainly via ACE and AT1 receptor inhibition [3,5]. Considering this, the antihypertensive potential of the extracts was evaluated according to their capacity for ACE inhibition.

The ACE inhibitory effect for EGP was 1.6× superior to the one found for GP, suggesting that the modification of the phenolic compound profile affects the capacity to interact with ACE; this is even though the enzymatic treatment was not observed to have a significant positive effect on the TPC or antioxidant capacity. This difference suggests that the ACE inhibitory potential is not only associated with the content of phenolic compounds, but also with their ability to interact with the enzyme, which is in accordance with the findings of Eriz et al. [52]. These authors studied the potential of ACE to inhibit anthocyanidins obtained from the skins and seeds of Chilean black grapes, obtaining a greater inhibition effect for the skin extract; however, the seed extract presented a greater phenolic content, which was attributed to the number of hydroxyl groups, the more frequent occurrence of epigallocatechin, and the lesser polymerization of proanthocyanidins in the skin extract.

In fact, an increase in phenolic acids, mainly caffeic acid, could contribute to this inhibition potential. Yu et al. [53] compared the antihypertensive potential of eleven phenolic acids and indicated that caffeic acid showed one of the best results. Agunloye and Oboh [54] also assayed caffeic and chlorogenic acids’ potential for ACE inhibition, reporting that caffeic acid inhibited almost 50% of ACE activity at a concentration of 25 µg/mL. Considering that the enzymatic extract showed a higher amount of caffeic acid, this could partly explain the better result obtained for the ACE inhibition using this extract.

Other phenolic modifications could also contribute to this inhibition potential. Montealegre et al. [55] studied the phenolic compounds found in the skins and seeds of different grape varieties, and reported that most flavonols are in their glucoside form in red grape skin. Considering this and the fact that phenolic compounds can be more bioactive when in their aglycone form, these results may be attributed to β-glucosidase activity, which can promote an increase in compounds in this form [27,29,30]. Nwanna et al. [56] studied different fruits from the *Solanum aethiopium* species and reported that the extract from *Solanum torvum* presented a better ACE inhibition effect, even though it was the extract with a lower phenolic content. The authors attributed this result to the greater amounts of certain types of phenolic compounds present in that extract, compared to the others.

More studies are necessary for a better understanding of the effect of enzymatic treatment on the phenolic structure of the extracts, which could better explain the results.

## 4. Conclusions

The use of a commercial maceration enzyme blend to assist in the process of extracting phenolic compounds from the red grape pomace, under the assay conditions, did not increase the levels of total phenolic compounds or the antioxidant capacity of the extracts obtained. However, the use of biotransformation for the deglycosylation of phenolic compounds has demonstrated its important ability to increase the bioactivity of flavonoids in diverse sources, as observed in a previous study using tannase on grape and orange matrices. Despite the fact that the enzyme cocktail did not present an increase in the phenolic content of the extracts obtained, the results regarding ACE inhibition using the enzymatic red grape extract employed in this work could indicate an alternative commercial enzyme for increasing the release of phenolic acids and promoting the biotransformation process for residues rich in glycosylated flavonoids.

A particularly important observation of this study is the great bioactive potential found in the extracts of these agro-industrial residues. Their antioxidant potential and phenolic content were highlighted, indicating that they are a good source of compounds, with potential for utilization as functional ingredients. The association between the antioxidant potential and the ACE inhibition potential demonstrates great possibilities regarding the application of these extracts in food or supplementation products, in order to assist in the control/treatment of individuals with arterial hypertension. The results can indicate the viability of upcycling this residue to obtain compounds of interest, thus reducing their environmental impact and promoting a circular economy. In-depth studies to better characterize the profile of bioactive compounds in this matrix and their biological potential are of great interest.

## Figures and Tables

**Table 1:** Extraction yield and TPC of extracts obtained from red grape pomace with and without enzymatic treatment.

Extract	Yield	TPC Extract	TPC Sample
	(%)	mg GAE/g DE	mg GAE/g DM
GP	24.5	52 ± 1 ^a^	13 ± 0.2 ^a^
EGP	40.3	54 ± 2 ^a^	22± 1 ^b^

Results are expressed as mean ± SD, except for yield values. Values followed by different letters in the same column present significant differences, according to Tukey’s test (*p* ≤ 0.1). mg GAE/g DE (dry extract). mg GAE/g DM (dry matter). GAE: Gallic Acid Equivalent. Extracts: GP—grape pomace; EGP—enzymatic grape pomace.

**Table 2:** Antioxidant capacity of extracts obtained from red grape pomace with and without enzymatic treatment, assessed using different methods.

Extract	DPPH	ORAC	FRAP
	(μmol TE/g DE)		
GP	644 ± 42 ^a^	2427 ± 84 ^a^	503 ± 43 ^a^
EGP	699 ± 62 ^a^	2461 ± 228 ^a^	485 ± 35 ^a^

Results are expressed as mean ± SD (*n* = 3). Values followed by same letters in the same column do not present significant differences according to Tukey’s test (*p* ≤ 0.1). Values are expressed as μmol TE/g DE (dried extract). TE: Trolox Equivalent. Extracts: GP—grape pomace; EGP—enzymatic grape pomace.

## Data Availability

Data is contained within the article.
